# Out-of-Hospital Cardiac Arrest As the Initial Presentation of Takotsubo Cardiomyopathy in the Presence of Severe Myocardial Bridge: A Diagnostic Challenge

**DOI:** 10.7759/cureus.110773

**Published:** 2026-06-13

**Authors:** Abeer Omar, Omar Makawi, Motaz Almahmood, Hibatallah Mohamed, Mawahib El Hassan

**Affiliations:** 1 Internal Medicine, Hamad Medical Corporation, Doha, QAT; 2 Cardiology, Hamad Medical Corporation, Doha, QAT; 3 Internal Medicine, Tower Health Medical Group Family Medicine - Phoenixville, Phoenixville, USA; 4 Medicine, University of Medical Sciences and Technology, Kigali, RWA

**Keywords:** instantaneous wave-free ratio (ifr), left anterior descending artery (lad), myocardial bridge (mb), out-of-hospital cardiac arrest (ohca), takotsubo cardiomyopathy (tc)

## Abstract

Takotsubo cardiomyopathy (TC) is characterized by transient, reversible systolic dysfunction in the absence of angiographic evidence of obstructive coronary artery disease (CAD). TC often mimics acute coronary syndrome (ACS) and is frequently precipitated by emotional or physical stress. In this report, a 45-year-old woman experienced out-of-hospital cardiac arrest (OHCA) due to ventricular fibrillation. Coronary angiography (CAG) identified severe myocardial bridging (MB), complicating the diagnostic process. Physiological assessment with instantaneous wave-free ratio (iFR) and fractional flow reserve (FFR) revealed no significant flow limitation attributable to MB. Transthoracic echocardiogram (TTE) demonstrated apical ballooning with reduced ejection fraction, which improved rapidly. These findings supported the diagnosis of TC. This case demonstrates that cardiac arrest (CA) can be the initial manifestation of TC. Clinicians should consider TC in patients presenting with CA or chest pain, particularly when reversible left ventricular dysfunction with apical ballooning is present. When MB coexists with TC, multimodality imaging and physiological assessment are critical for accurate diagnosis.

## Introduction

Takotsubo Cardiomyopathy (TC), or stress-induced cardiomyopathy, is a reversible myocardial injury marked by transient left ventricular systolic dysfunction without obstructive coronary artery disease (CAD) [1]. TC commonly presents with chest pain, dyspnea, and electrocardiographic changes that resemble acute coronary syndrome (ACS). It is typically associated with acute stressful events, including unexpected bereavement, interpersonal conflict, or physical stress. Although TC is generally reversible, it can be associated with severe complications such as cardiogenic shock, arrhythmias, and, in rare cases, cardiac arrest (CA) [2].

In a registry by Gili et al., CA occurred in 5.9% of TC patients and was linked to a six-fold increase in both short- and long-term mortality [3]. Previous studies indicate that TC accounts for approximately 1-2% of patients presenting with troponin-positive suspected ACS or suspected ST-elevation myocardial infarction (MI). TC predominantly affects post-menopausal women [4-6].

Myocardial bridge (MB) is a congenital coronary anomaly in which a segment of a coronary artery runs intramyocardially. While typically benign, it has been associated with myocardial ischemia, arrhythmias, and sudden cardiac death [7,8]. Both TC and MB are rare conditions, and literature regarding their simultaneous occurrence is limited and sometimes conflicting [9,10]. The coexistence of TC and MB presents a diagnostic challenge, particularly when both conditions may contribute to myocardial ischemia or arrhythmia.

This case report presents a rare case of TC manifesting as out-of-hospital cardiac arrest (OHCA) in the context of severe MB.

## Case presentation

A 45-year-old premenopausal woman with a history of multinodular goiter (status post left hemithyroidectomy) and prior gastric sleeve surgery presented with severe chest pain and shortness of breath, followed by OHCA due to ventricular fibrillation (VF). Cardiopulmonary resuscitation (CPR) was initiated by a family member and continued by emergency medical services. The patient received nine defibrillation shocks with a total CPR duration of 30 minutes before achieving a return of spontaneous circulation. On admission, her heart rate was 96 beats per minute (bpm; reference range: 60 to 100 bpm), blood pressure was 129/73 mmHg (reference range: 90/60 to 120/80 mmHg, respiratory rate was 26 breaths per min (reference range: 12 to 18 (or up to 20 breaths per min), and oxygen saturation was 96% (reference range: 94%-98%) on mechanical ventilation. Physical examination revealed normal heart sounds, no murmurs, good bilateral air entry, and cold, clammy extremities. She was admitted to the cardiac intensive care unit (ICU) for further management.

Laboratory findings showed elevated troponin, mildly elevated pro-B-type natriuretic peptide (pro-BNP), and lactic acidosis. Renal and thyroid function tests were normal (Table 1).

**Table 1:** Laboratory values on the initial day of presentation and two days later T-HS: Troponin high-sensitivity; Pro-BNP: pro B-type natriuretic peptide; TSH: Thyroid stimulating hormone; PO2: Partial Pressure of Oxygen; PCO2: Partial pressure of carbon dioxide.

Laboratory marker	Initial day of presentation	Two days later	Normal reference range
Serum levels			
Troponin (T-HS)	344 ng/L (AM) > 1507 ng/L (PM)	375 ng/L	Males < or = 15 ng/L; Females < or = 10 ng/L
Pro BNP	231 pg/mL	2626 pg/mL	<300 pg/mL
Urea	5.9 mmol/L	5.2 mmol/L	2.9-7.1 mmol/L
Creatinine	87 µmol/L	52 µmol/L	Male: 62-115 µmol/L; Female: 44-97 µmol/L
Bicarbonate	15 mmol/L	22 mmol/L	23-28 mmol/L
TSH	1.6 mU/L	-	0.5-4.0 mU/L
T4	215.0 nmol/L	-	64.4-154.4 nmol/L
Venous blood gases			
pH initial	7.00	7.32	7.32-7.41
PO2	58 mmHg	52 mmHg	35-42 mmHg
PCO2	70 mmHg	50 mmHg	42-53 mmHg
Bicarbonate	-	26 mmol/L	24-28 mmol/L
Lactate	7.5 mmol/L	0.7 mmol/L	0.5-2.2 mmol/L
Glucose	19.7 mmol/L	5.9 mmol/L	4.1-5.9 mmol/L
Arterial blood gases			
pH initial	7.25	7.41	7.38-7.44
PO2	78 mmHg	212 mmHg	75-100 mmHg
PCO2	52 mmHg	39 mmHg	38-42 mmHg
Bicarbonate	23 mmol/L	25 mmol/L	23-26 mmol/L
Lactate	3.2 mmol/L	0.6 mmol/L	0.5-2.2 mmol/L
Glucose	14.6 mmol/L	5.4 mmol/L	4.1-5.9 mmol/L

Electrocardiography showed a normal sinus rhythm with a heart rate of approximately 100 beats per minute, T-wave inversion in lead V3 without ST-segment elevation (Figure 1).

**Figure 1:**
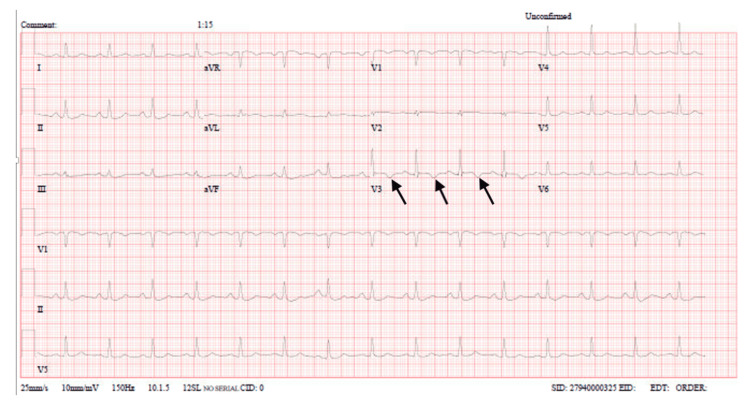
Electrocardiogram Electrocardiography showed normal sinus rhythm with heart rate approximately 100 beats per minute, T-wave inversion in lead V3 without ST-segment elevation.

Chest X-ray showed bilateral infiltrates with pulmonary congestion. Transthoracic echocardiogram (TTE) revealed a dilated left ventricle, regional wall motion abnormalities, and a left ventricular ejection fraction (LVEF) of 37% with an apical ballooning pattern (Video 1).

**Video 1:** TTE showing apical ballooning with low LVEF TTE: Transthoracic Echocardiogram; LVEF: Left Ventricular Ejection Fraction.

Repeat TTE after 48 hours showed improvement of LVEF to 55% (Video 2).

**Video 2:** TTE showing improvement in LVEF (normal LVEF) after two days TTE: Transthoracic Echocardiogram; LVEF: Left Ventricular Ejection Fraction.

Coronary angiography (CAG) demonstrated mild disease in the left anterior descending artery (LAD) and severe MB (Figure 2).

**Figure 2:**
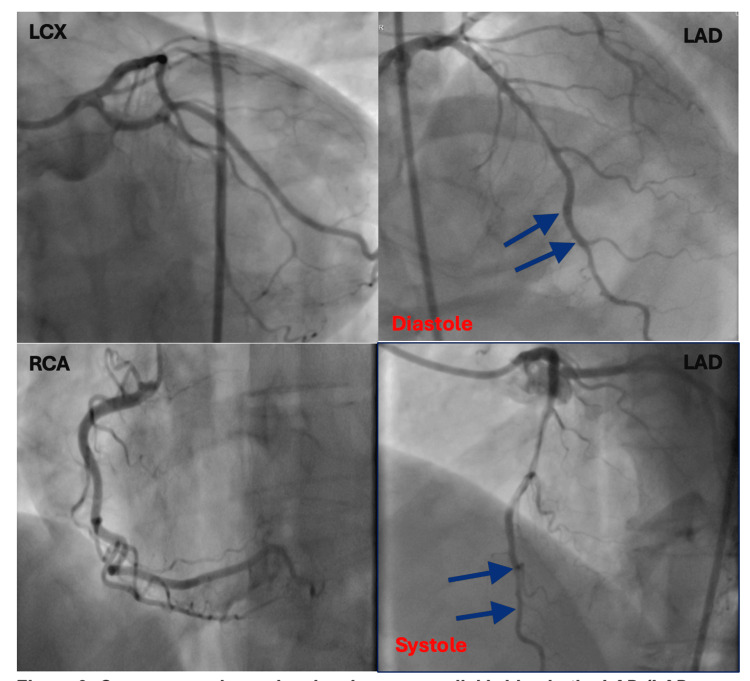
CAG showing myocardial bridge in the LAD (LAD artery narrowing during systole) CAG: coronary angiography; LAD: left anterior descending; RCA: right coronary artery; LCX: left circumflex.

Further physiological assessment showed an initial instantaneous wave-free ratio (iFR) of 0.28 during coronary spasm, a repeat iFR of 0.92 after nitrate administration, and a fractional flow reserve (FFR) of 0.80, indicating no significant flow limitation due to MB. Intravascular ultrasound (IVUS) was performed on the LAD, showing a systolic LAD minimal luminal area (MLA) of 8.2 mm^2^ in the bridging area (not significant) and a diastolic LAD MLA of 11.2 mm^2^ in the bridging area (not significant).

Cardiac magnetic resonance imaging (MRI) performed weeks later demonstrated normal ventricular function with no evidence of MI, edema, fibrosis, or myocarditis. The delayed timing of imaging likely limited its sensitivity in detecting acute takotsubo changes.

The patient was initially managed with dual antiplatelet therapy, which was later discontinued after the exclusion of obstructive CAD. She was started on a calcium channel blocker (diltiazem) and received supportive ICU care, including mechanical ventilation, which was subsequently weaned. Her hospital course was complicated by hypoxic-ischemic encephalopathy, tetra paresis, aspiration pneumonia, and delirium, all of which improved with appropriate management. She underwent intensive rehabilitation, such as physiotherapy and occupational therapy, along with neuropathic pain management. Given the presentation with VF- CA, an implantable cardioverter-defibrillator was placed for secondary prevention.

At her one-month follow-up, the patient was alert, fully oriented, and hemodynamically stable on room air. Neurological assessment showed gradual improvement with upper limb power of 3/5 and lower limb power of 2/5. With continued rehabilitation, she regained independent ambulation.

## Discussion

This case report details an OHCA arrest in a 45-year-old premenopausal patient without chronic comorbidities, who was diagnosed with both TC and MB. Emotional triggers included the sudden death of her father and a house fire. The coexistence of MB and TC introduced diagnostic uncertainty; however, physiological assessment (FFR/iFR) revealed no significant flow-limitation, suggesting transient vasospasm rather than fixed obstruction. Rapid improvement in LVEF and the presence of a characteristic apical ballooning pattern supported the diagnosis of TC. Cardiac MRI performed later in the disease course was normal, consistent with resolution of transient myocardial dysfunction, underscoring the importance of timely imaging. This case highlights the value of multimodality imaging and physiological assessment (FFR/iFR) in differentiating between competing diagnoses in complex clinical scenarios.

The concurrence of TC and MB represents a noteworthy addition to the literature. Previous reports have documented the coexistence of TC and MB [11], and another case highlighted the association between MB and TC [12]. In a study by Lemaitre et al., MB possibly enhanced by catecholamines during stress may, in association with left ventricular hypertrophy, contribute to the preferential apical localization observed in the apical ballooning syndrome [13]. Although the precise etiology of TC is not known, the most plausible causes of TC are catecholamine excess, microvascular dysfunction, and myocardial stunning [14]. 

Sudden CA may be the first manifestation of TC [15]. A study by Syed et al. estimated the frequencies of various arrhythmias in TC, including a prevalence of 2.2% for VF, 1.2% for sustained ventricular tachycardia, 1% for non-sustained ventricular tachycardia, 0.5% for asystole, 2.9% for atrioventricular block, 1.3% for sinus node dysfunction, 4.7% for atrial fibrillation, and 1.1% for sudden cardiac death. Ventricular arrhythmias leading to OHCA occurred in 1.1% of Takotsubo cases [16].

TC predominantly affects post-menopausal women with emotional or physical risk factors. Our patient was premenopausal, which is atypical. Previous reports have described TC with unusual presentations with and without MB. One case report highlighted TC in a 76-year-old woman without identifiable triggers; however, there was no MB or CA [17]. Another report described a 31-year-old healthy woman with emotional triggers who presented with OHCA; however, there was no MB [18]. Dugal et al. reported a case of mid-ventricular variant TC with a coexisting LAD MB in a 60-year-old elderly woman with multiple comorbidities and no identifiable emotional triggers. There was no CA presentation [19]. As per our PubMed search, none of the previous case reports or studies include a presentation with all three elements of TC, MB and CA combined together. The occurrence of CA in a patient with both TC and MB represents a notable and uncommon clinical scenario.

Socioeconomic factors such as education, income, employment, and living conditions, along with mental health conditions including depression and anxiety, significantly influence the development and progression of cardiovascular diseases. Lower socioeconomic status and pre-existing mental health disorders are associated with elevated cardiovascular risk [20]. Teddy et al. demonstrated that both rural hospital location and low socioeconomic status independently correlated with increased in-hospital mortality among patients admitted with TC [21]. These findings underscore the necessity of addressing socioeconomic determinants and mental health conditions to mitigate cardiovascular disease risk.

## Conclusions

This case report presents a unique case of TC manifesting as OHCA in the context of severe MB, creating a diagnostic challenge. Clinicians should consider TC in patients presenting with CA or chest pain, particularly when reversible left ventricular dysfunction with apical ballooning is present. In cases of coexisting MB, a thorough physiological evaluation (FFR/iFR) is crucial to accurately identify the underlying cause of ischemia or arrhythmia. Further research is needed to clarify the association between MB and TC. Comprehensive socioeconomic data should be integrated into cardiovascular health strategies.
